# Can alpine plant species “bank” on conservation?: Using artificial aging to understand seed longevity

**DOI:** 10.1002/aps3.11493

**Published:** 2022-09-30

**Authors:** Alexandra E. Seglias

**Affiliations:** ^1^ Department of Research and Conservation Denver Botanic Gardens, 909 York Street Denver, Colorado 80206 USA

**Keywords:** accelerated aging, alpine, conservation, germination, seed banking, viability

## Abstract

**Premise:**

To conserve native plants, many institutions are turning toward ex‐situ conservation methods, such as storage in seed banks; however, not all seeds are able to survive in seed bank conditions, or may not in the long term. Experimental aging has shown that alpine species lose viability more quickly than low‐elevation species. Furthermore, the germination requirements for rare species are largely unknown, but are a necessary first step in understanding storage behavior and viability decline.

**Methods:**

Five alpine species were subjected to germination and accelerated aging experiments to understand their longevity in storage. For the accelerated aging experiment, the seeds were rehydrated in a dark incubator and subsequently placed in a drying oven. Following the aging process, the seeds were placed into previously determined germination conditions.

**Results:**

All species had *p*_50_ values of <13.7 days, which is the threshold to consider a species short lived. These results suggest that we cannot haphazardly store seeds and assume that all species will survive for decades.

**Discussion:**

Accelerated aging experiments are not a perfect measure of seed longevity, and true longevity needs to be empirically determined. However, this experimental method allows us to predict which species may be short lived and whether alternative ex‐situ conservation methods might be needed beyond conventional seed banking.

Climate change threatens plant biodiversity worldwide. Alpine plant species are particularly vulnerable to climate change, as temperature fluctuations are projected to be most severe at high elevations. Species will be displaced to higher and higher elevations, or highly restricted microsites, until there is nowhere else to go (Intergovernmental Panel on Climate Change, 2014). High‐elevation areas are warming faster than the global average (Pepin et al., 2015); for example, in the Rocky Mountains of Colorado, USA, the snowmelt has advanced 4.8 days on average per decade from 1978–2007, and the November–May air temperatures have increased by a median of 0.9°C per decade (Clow, 2010). Even small shifts in climate can have major consequences on plant phenology, plant–pollinator interactions, reproduction, fitness, and community composition. Furthermore, the limited space for plant species to migrate upward to track a suitable climate coupled with potential trait or phenological changes may increase their extinction risk.

To conserve native plant species at risk from climate change and a multitude of other factors, many institutions are turning toward ex‐situ methods when in‐situ conservation may not be feasible or sufficient for the long term. Worldwide, seed banks have become a primary method of ex‐situ conservation due to their high resource efficiency and low loss of genetic diversity (Havens et al., 2014). Crops and other economically important species have been the primary focus of seed storage, but with the adoption of the Global Strategy for Plant Conservation in 2002, the importance of seed storage for the conservation of wild species has been realized (Hay and Probert, 2013). Seed banks store seeds under low moisture (relative humidity of 15% ± 3%) and temperature (−18°C ± 3°C) conditions (FAO, 2014), which slow the rate of deterioration and the loss of viability. Seed banks allow the preservation of orthodox (desiccation‐tolerant) seeds for up to hundreds of years and are a great option for those that can survive drying and below‐freezing conditions.

However, an estimated 10,000 species of plants need alternative methods of ex‐situ conservation, as they are desiccation‐sensitive (recalcitrant) or produce few to no seeds to build a seed collection for storage (Pence, 2010). These species are deemed “exceptional” and cannot be propagated by seed or stored using traditional seed banking methods (Pence, 2011; Fant et al., 2016). Furthermore, there are an estimated tens of thousands of species that are likely to be short lived in seed banks (time for seed lot to fall to 50% viability of ≤20 years in conventional seed banking conditions; Pence et al., 2022). Species falling into this category may also be considered exceptional and require either alternative methods of conservation or methods to augment traditional seed banking, such as cryopreservation or tissue culture. Empirical seed storage research has not been completed for the majority of plant species in the world, but there have been studies to show that we can make predictions of storage longevity based on family, genus, life history, and/or habitat of origin (e.g., Walters et al., 2005; Mondoni et al., 2011; Salazar et al., 2018; Satyanti et al., 2018; Tausch et al., 2019). For example, alpine species in Italy and Australia have shown shorter longevity than species from lower elevations (Mondoni et al., 2011; Satyanti et al., 2018), while seeds from species sourced from warmer and more arid environments survived longer in storage (Walters et al., 2005; Probert et al., 2009).

Many seeds are expected to survive in ex‐situ seed banks for hundreds of years, which means viability loss might not be seen for decades (Walters et al., 2005), making seed storage longevity studies challenging. A way to study seed bank longevity more quickly is to use artificial aging methods, which accelerate the loss of seed viability using warm and humid conditions over a short period of time (Newton et al., 2009). Although the conditions in seed banks are vastly different from those used in artificial aging tests, genera and families that have been shown to be short lived in aging tests have also shown evidence of being short lived in long‐term storage at −20°C (Probert et al., 2009). As such, although they provide an indirect prediction that needs to be confirmed with empirical tests, artificial aging experiments are the best option for quickly estimating seed longevity. This method is robust, standardized, and has been adapted in recent studies of seed longevity of wild species to better understand how to conserve species from specific habitats or regions (e.g., Mondoni et al., 2011; Satyanti et al., 2018; Tausch et al., 2019). The analyses for aging tests use the time taken for seed viability to fall to 50% (*p*_50_) as an index to predict storage longevity. Understanding seed longevity is essential for effective ex‐situ conservation through seed banking. This is particularly true for rare and threatened species when climate change, human disturbance, and other factors threaten the survival of the species and populations may not be available in the future from which to re‐collect seed.

The Research and Conservation Department at Denver Botanic Gardens (the Gardens; Denver, Colorado, USA) partners with the Center for Plant Conservation (Escondido, California, USA) to monitor and collect seed from over 70 rare plant species across Colorado and the broader southwestern region. As part of the North American Botanic Gardens Strategy for Alpine Plant Conservation (Ripley et al., 2020), the Gardens has a large focus on the alpine plants of Colorado, particularly those considered threatened or rare. Understanding the germination requirements, storage behavior, and optimal methods for long‐term storage is essential for effectively conserving threatened alpine species. This study aims to elucidate the germination requirements and seed longevity of alpine species in Colorado, specifically rare alpine species. If Colorado alpine species are short lived, like alpine species from other parts of the world, we can further determine how alpine environments may influence seed longevity and how to better conserve these at‐risk species.

## METHODS

### Characteristics of study area

Seeds were collected from populations located in the Mosquito Range of the Colorado Rocky Mountains, USA. The range trends north to south in central Colorado, bordered by the upper Arkansas River Valley and the Sawatch Range to the west, and South Park and the southern Front Range to the east. The climate is characterized as continental, with mean annual temperatures ranging from about 2°C at elevations around 3000 m to −5°C at the highest elevations above 4000 m. The mean annual precipitation occurs mostly as snow and is ~76 cm on average across the range, but varies from ~40 cm at lower elevations to ~120 cm on high peaks (Brugger et al., 2019).

### Species and population sampling

**Figure 1:**
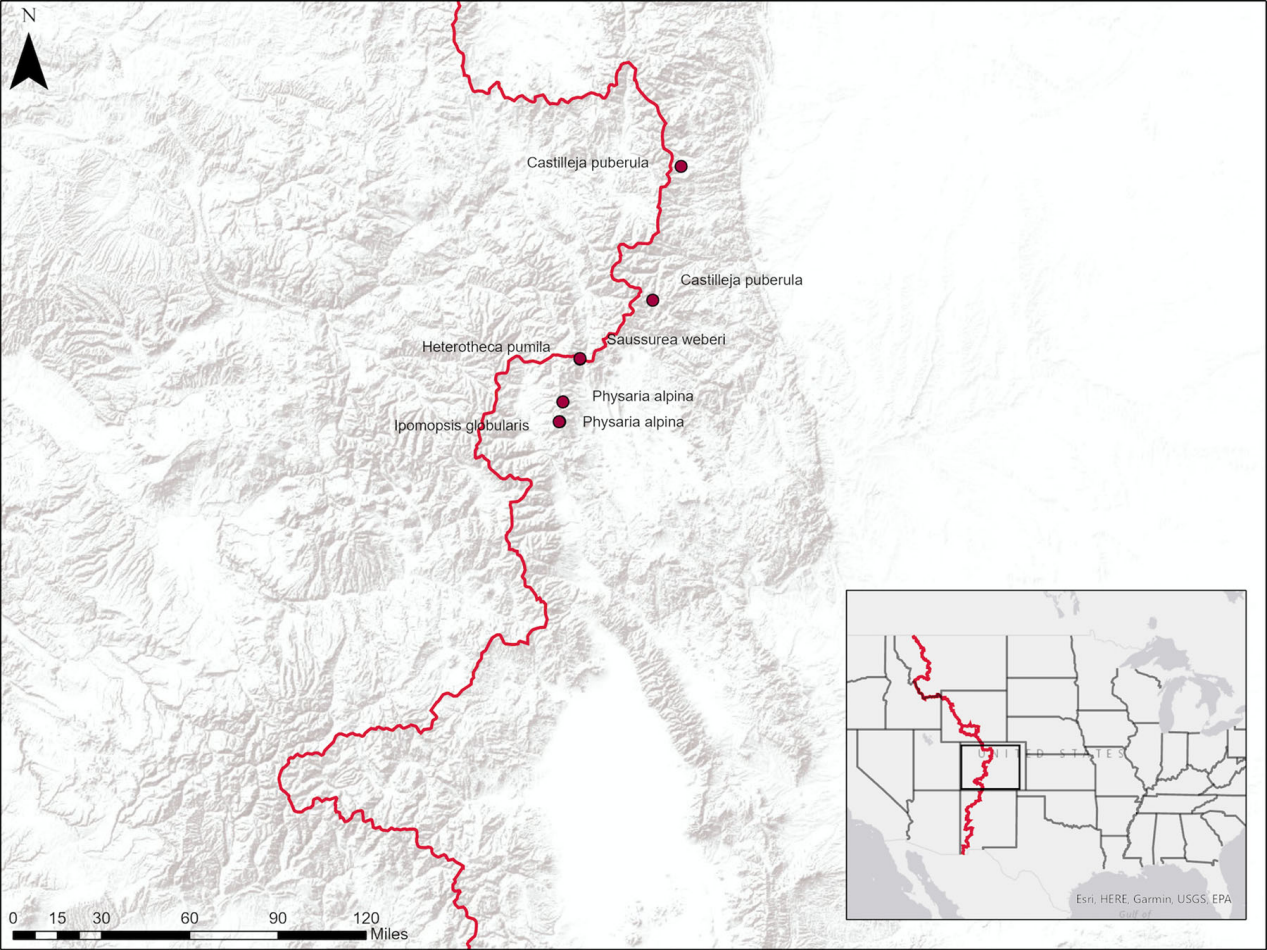
Seed collection locations in the Rocky Mountains of Colorado, USA. The points overlap for *Heterotheca pumila* and *Saussurea weberi* and the southern population of *Physaria alpina* and *Ipomopsis globularis*. The red line denotes the Continental Divide, which relates to the high elevation in the state.

Five native forb species with differing levels of rarity (according to NatureServe [2022] Global [G] rankings) were selected for this study (Table 1): *Castilleja puberula* Rydb. (Orobanchaceae), *Heterotheca pumila* (Greene) Semple (Asteraceae), *Ipomopsis globularis* (Brand) W. A. Weber (Polemoniaceae), *Physaria alpina* Rollins (Brassicaceae), and *Saussurea weberi* Hultén (Asteraceae). Two populations were evaluated for *C. puberula*, as it is important to examine population‐level variation in germination (e.g., Seglias et al., 2018), but multiple populations were not available for all species. Seeds were collected from wild populations in 2018 or 2019 in alpine regions of Colorado (Figure 1). Populations were located using Element Occurrence Records provided by the Colorado Natural Heritage Program (received in 2014). Seed collection was limited to 10% of the total mature seed of the population for the species considered rare (G3 rank and under) and 20% for *H. pumila*, and seed was collected from at least 40 plants for all species. Vouchers were collected from each population and deposited in the Kathryn Kalmbach Herbarium (KHD) at Denver Botanic Gardens (Appendix 1). The seeds were cleaned and kept at room temperature until the start of the experiment (about 5–7 months). No known germination requirements exist for the rare species (*H. pumila* has germination information in Kew's Seed Information Database; Royal Botanic Gardens Kew, 2021). Data on congeners of these species provide the following information on dormancy classification: *Castilleja* – physiological dormancy (requiring a period of cold stratification to break dormancy; PD); *Heterotheca* – no dormancy (does not need to break dormancy and can germinate under a wide range of temperature conditions; ND); *Ipomopsis* – PD; *Physaria* – no information; *Saussurea* – PD or ND (Baskin and Baskin, 2014). The dormancy classification of congeners, coupled with climatic information of the source habitat, provides information on potential dormancy breaking and germination requirements.

**Table 1:** Collection information for the study species. G‐Rank data are globally applied conservation rankings (NatureServe, 2022) showing state‐level data for Colorado, USA.

Species	Latitude	Longitude	Elevation (m)	Collection site	County	G‐Rank
*Castilleja puberula* a	40.0973	−105.5858	3451	Mt. Audubon	Boulder	G3/S2
*Castilleja puberula*	39.5924	−105.7247	3566	Guanella Pass	Clear Creek	
*Heterotheca pumila*	39.3699	−106.0803	3699	Hoosier Pass	Summit	G4/S4
*Ipomopsis globularis*	39.1315	−106.1823	3639	Weston Pass	Park	G2/S2 (endemic)
*Physaria alpina* b	39.2072	−106.166	3672	Fourmile Creek Trail	Park	G2/S2 (endemic)
*Physaria alpina*	39.1304	−106.1837	3613	Weston Pass	Park	
*Saussurea weberi*	39.3706	−106.0816	3699	Hoosier Pass	Summit	G3/S2

^a^ Both populations of *Castilleja puberula* were used in the germination experiment, but only the Guanella Pass population was used in the longevity experiment.

^b^ The Fourmile Creek population of *Physaria alpina* was used in the germination experiment but the population from Weston Pass (collected in 2019) was used in the longevity experiment due to a low number of seeds following the first experiment.

### Germination experiment

Immediately before initiating germination treatments, the seeds were sterilized in 0.25% sodium hypochlorite (bleach) solution for 30 s and then rinsed two times in sterile deionized water. Seeds were placed in 47‐mm sterile plastic Petri plates containing 1.5% agar. Each plate consisted of 16 seeds (*C. puberula*, *H. pumila*, and *I. globularis*) or nine seeds (*P. alpina* and *S. weberi*, due to limited seed quantity), with four replications per treatment. Seeds were subjected to dark, moist stratification (2–3°C) for two, six, and 12 weeks prior to incubation, or no stratification (control treatment). The seeds were monitored throughout stratification to check germination. Following stratification, the seeds were placed in two incubation treatments (15/6°C and 20/10°C, with an alternating 12 h light/12 h dark regime) in climate‐controlled incubators (Intellus Environmental Controller; Percival Scientific, Perry, Iowa, USA) for two weeks. Germination was evaluated every 48 h during the incubation. Seeds with radicle emergence were considered to have germinated and were removed from the Petri plate (Baskin and Baskin, 2014). Seeds that did not germinate by the end of the treatment were removed and subjected to a “cut test,” which is a common practice to determine viability of seeds at the end of a germination experiment. Seeds that had an intact embryo were considered viable. Because these are rare species, the ungerminated seeds were transferred to germination boxes and incubated at 20/10°C to increase the potential for germination and to not waste seeds. Seedlings were given to the greenhouse staff and planted in the living collection at the Gardens or Betty Ford Alpine Garden in Vail, Colorado.

### Longevity methods

*Ipomopsis globularis* was not used in the accelerated aging experiment due to its low germination, the uncertainty of its germination requirements, and the inability to collect more seeds to attempt different germination treatments prior to the longevity experiment. Only the Guanella Pass population of *C. puberula* was used for the longevity experiment, as its germination percentages were higher.

Accelerated aging tests were performed using the protocol for comparative seed longevity testing (Newton et al., 2009; Probert et al., 2009). This method exposes seeds to warm and humid conditions to expeditiously decrease seed viability through two main processes: rehydration and aging. To rehydrate the seeds, 10 samples (seven samples for *S. weberi* due to limited seed quantity) of 50 seeds for each species were placed in open glass Petri plates inside an airtight electrical enclosure box above an unsaturated solution of LiCl (385 g, anhydrous in 1 L of distilled water). The box was placed in an incubator with constant light at 20°C for two weeks. These conditions created a relative humidity of 47%. Following rehydration, the seeds were placed in the aging conditions, which consisted of 300 g LiCl in 1 L of distilled water inside an enclosed electrical box inside a dark drying oven set to 45°C. These conditions created a relative humidity of 60%. The relative humidity was checked weekly using a hygrometer and adjusted with distilled water as necessary.

One Petri plate of 50 seeds for each species was removed for germination testing at each time interval (one, three, five, 10, 17, 25, and 35 days; *Saussurea weberi*: one, three, five, and 25 days). Seeds were sown on agar in Petri boxes and placed in an incubator under previously determined germination conditions. Germination was scored every 2–3 days for five weeks.

### Statistical analysis

All analyses were performed on viability‐adjusted germination rates (number of germinated seeds/number of viable seeds) using the R environment for statistical computing (R Core Team, 2022). Generalized linear models (GLMs) with binomial error and logit link function were fitted to the final germination results for all species together, and for each species individually, using stratification length and incubation temperature as predictors. Population was used as an additional predictor for *C. puberula*, as seed was collected from two separate populations for this species. Maximal models for all species included two‐way interactions among predictors. The best‐fit model was determined with the Akaike information criterion (AIC) using the ‘aictab’ function from the AICcmodavg package (Mazerolle, 2020).

For the accelerated aging experiment, a probit analysis was performed with the ‘drc’ package (Ritz and Strebig, 2016). This analysis estimates the time for the seed viability to fall to 50% (*p*_50_) by fitting the viability equation (Ellis and Roberts, 1980):

$$v=K_{i}-(p/\sigma )$$

where *v* is the viability in normal equivalent deviates (NED) at *p* days in storage, *K*_*i*_ is the estimated initial viability, and σ is the standard deviation of the normal distribution of seed deaths over time.

## RESULTS

### Germination requirements

**Figure 2:**
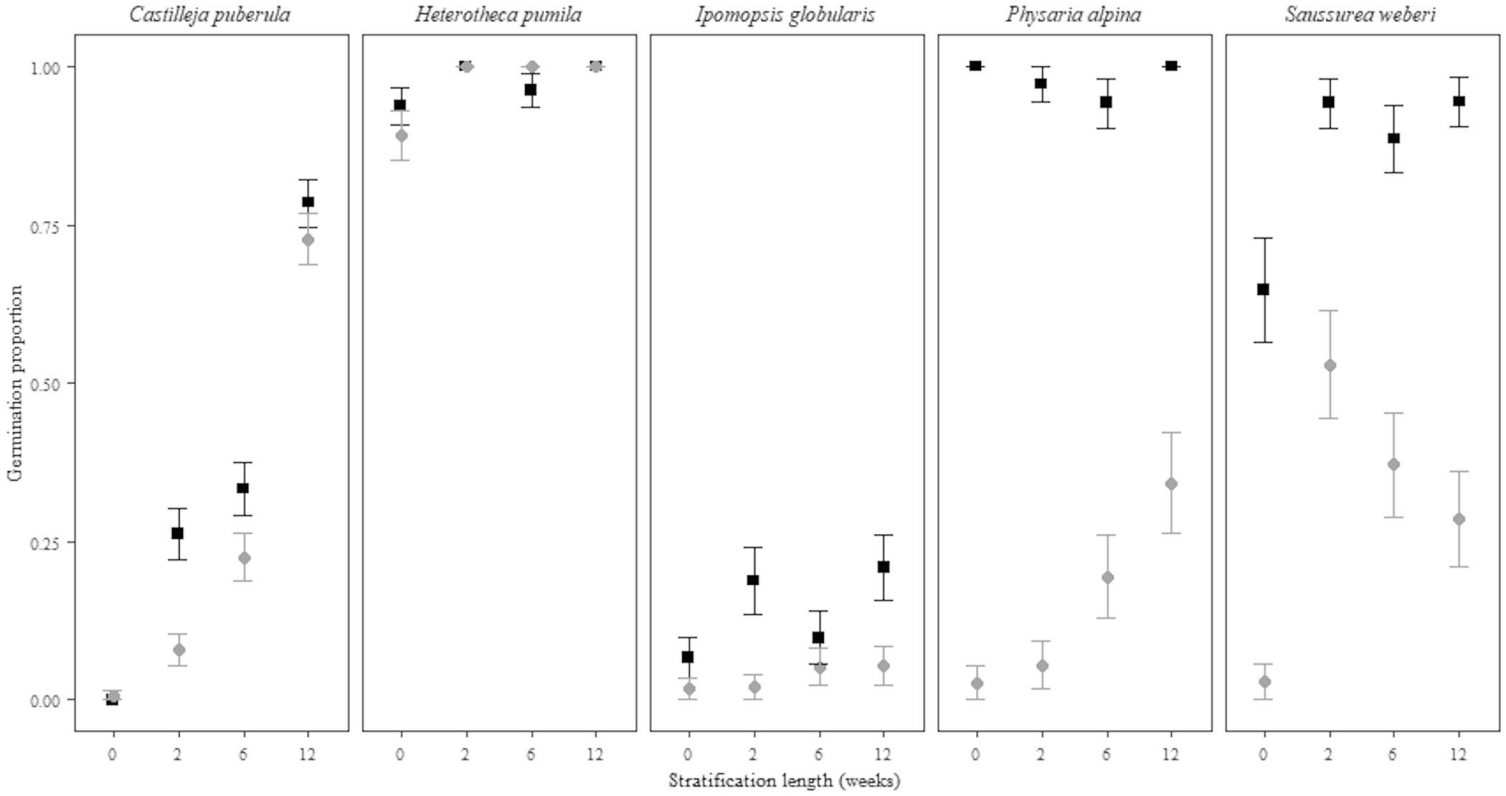
Final germination proportion for each species by treatment. The populations of *Castilleja puberula* are combined. The results are based on the best‐fit GLM with treatment factors as predictors (binomial error with logit link function). Gray dots denote the cool incubation temperature (15/6°C), and black squares denote the warm incubation temperature (20/10°C). Standard error bars are displayed.

**Figure 3:**
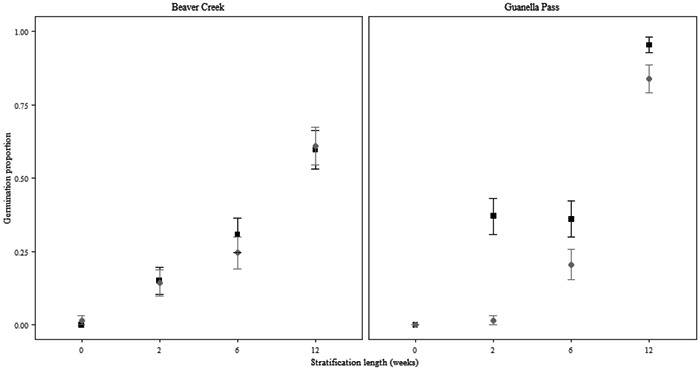
Final germination proportion for the two populations of *Castilleja puberula*. The results are based on the best‐fit GLM with treatment factors as predictors (binomial error with logit link function). Gray dots denote the cool incubation temperature (15/6°C), and black squares denote the warm incubation temperature (20/10°C). Standard error bars are displayed.

The germination rates significantly differed among species and treatments (*P* < 0.001; Figure 2; Appendix S1). Stratification length was significant for *C. puberula*, *H. pumila*, and *S. weberi* (*P* < 0.001), although the differences in germination rate were not biologically relevant for *H. pumila* (this genus is known to be nondormant and had a germination of >80% in all treatments). Both populations of *C. puberula* germinated to the highest percentages in the 12‐week stratification treatment (Figure 3). Incubation temperature was more important for the population from Guanella Pass, whereas the population from Beaver Creek had similar germination rates under both incubation treatments. Additionally, high germination occurred during the stratification period, which must be considered more in relation to its ecological implications (see Seglias et al., 2018). *Saussurea weberi* had significantly higher germination in treatments with at least two weeks of stratification followed by warm incubation conditions (*P* < 0.001; Figure 2).

Incubation temperature had a significant effect on the germination of *I. globularis* (*P* < 0.01) and *P. alpina* (*P* < 0.001). *Ipomopsis globularis* had low germination across all treatments, but its germination in the warm incubation treatment was slightly higher than in the cool incubation (Figure 2). Additionally, some stratification was beneficial, but germination did not significantly differ following the different stratification treatments. More experiments are needed for this species to determine its optimal germination conditions, such as a longer stratification period. *Physaria alpina* germinated on average close to 100% in the warm incubation treatment, but its responses to the stratification lengths experienced before incubation were not significantly different (*P* = 0.18; Figure 2).

### Storage longevity

**Figure 4:**
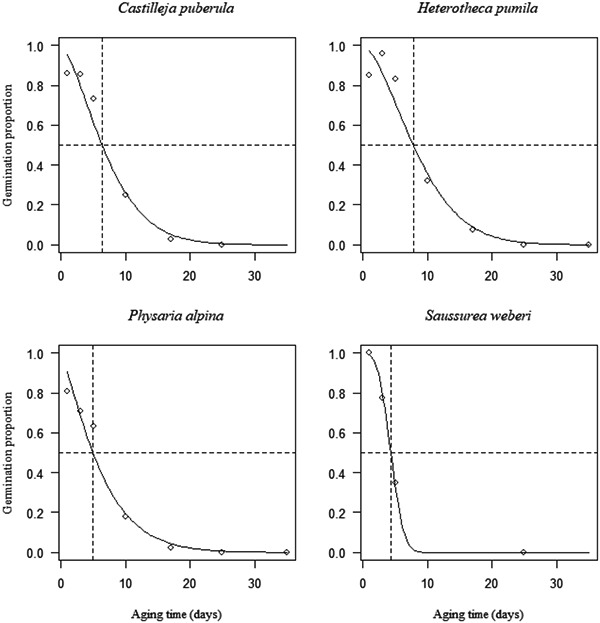
Seed survival curves in artificial aging conditions at 45°C and a relative humidity of 60%. The curves were fitted using a probit analysis (solid lines). Open dots show the germination proportion on each day of aging, and dashed lines show when germination fell below 50% (*p*_50_) on the given day of aging.

Seed viability quickly declined during experimental aging for all seed lots (Appendix S2). All species had *p*_50_ (time to 50% germination) values less than 13.7 days (Figure 4), which was the threshold to consider a species short lived (Probert et al., 2009; Satyanti et al., 2018). Other studies take an even more conservative approach and define short‐lived species as those with a *p*_50_ between 1 and 10 days (Mondoni et al., 2011; Tausch et al., 2019). *Physaria alpina* and *S. weberi* had *p*_50_ values less than five days (4.9 and 4.3 days, respectively). *Castilleja puberula* had a *p*_50_ of 6.4 days, and *H. pumila* had the longest *p*_50_ of the species at 7.8 days. These lower *p*_50_ values further confirm the finding that these species are short lived.

## DISCUSSION

Germination is a critical life stage for many plant species. Knowing the conditions that species need to germinate is crucial for scientists to successfully grow species for restoration projects and understand viability loss over time in ex‐situ storage; however, for rare and threatened species, a broad understanding of their biology and reproduction, including their germination requirements, is often lacking (Godefroid et al., 2010). Prior to this study, the germination requirements for four of these five alpine species were not known and/or documented (*H. pumila* has germination information in the Seed Information Database; Royal Botanic Gardens Kew, 2021). Four of the five species (*C. puberula*, *H. pumila*, *P. alpina*, and *S. weberi*) germinated to high percentages (>75%) in at least one of the treatments, which provides us with necessary information to effectively conserve these species.

The species in this study showed a mix of ND and PD. Of the alpine species for which germination and dormancy information is available, about 24% and 49% of alpine forbs exhibit ND and PD, respectively (Baskin and Baskin, 2014), with PD being the predominant dormancy mechanism (of the five dormancy classifications) of alpine species (Schwienbacher et al., 2011; Fernández‐Pascual et al., 2020). *Heterotheca pumila* showed evidence of ND, as this species germinated to very high percentages without a period of stratification. Consistent with this, *Hetherotheca* as a genus has been shown to have ND (Baskin and Baskin, 2014). The dormancy of *P. alpina* is not as clear. There is some evidence that the species may be ND or has non‐deep PD, whereby dormancy was broken during the after‐ripening period or with high temperatures during incubation. Two species of other *Physaria* have been shown to have PD (Baskin and Baskin, 2014). When testing seed viability or growing plants for conservation projects, a period of stratification may not be necessary for either of these species, but *P. alpina* needs high incubation temperatures to germinate to high proportions, which has been shown for other alpine species as well (Fernández‐Pascual et al., 2020). *Castilleja puberula* and *S. weberi* both display PD, as some period of stratification was necessary to germinate to high proportions. Both of these genera have been previously shown to have PD (Baskin and Baskin, 2014). *Castilleja puberula* needs at least 12 weeks of cold stratification for high levels of germination, while *S. weberi* will germinate to close to 100% after only two weeks of stratification. These conditions should be considered in conservation actions that involve seed germination.

The germination information gleaned from this and other studies can be applied to other alpine species or lowland congeners for which information may be lacking. In general, stratification and alternating temperatures during the incubation period are known to increase the final germination proportion of alpine species (Fernández‐Pascual et al., 2020). Similar to the results of this study, temperatures above 20°C during the incubation period had a positive effect on the germination of strict alpine species in Italy, with 79% of the study species showing optimal germination in these conditions (Mondoni et al., 2011). In trying to germinate alpine species with unknown dormancy breaking and germination requirements, the above conditions can be used as a starting point.

Under artificial aging conditions, the seed longevity (*p*_50_) of the Colorado alpine species in this study ranged from 4.3 to 7.8 days. Although we cannot extrapolate an exact time period for which these species may survive in ex‐situ seed bank conditions, accelerated artificial aging tests are the best method for applying a standardized protocol to quickly test the longevity of a wide range of genera. Although the conditions are not the same as those the seeds will experience in traditional ex‐situ seed banking conditions, there is evidence that artificial aging can predict seed viability loss during long‐term storage across numerous plant families. In a study comparing the results of artificial aging to those of storage after 20 years in a seed bank, 46 families showed a significant correlation in their viability decline in both conditions (Probert et al., 2009). The results from the current study suggest that all five species should be considered short lived (*p*_50_ under both the 13.7‐day [Probert et al., 2009] and 10‐day [Mondoni et al., 2011] thresholds), which is consistent with previous studies of alpine plants in Italy and Australia (Mondoni et al., 2011; Satyanti et al., 2018). These results correspond with studies that have shown that seeds sourced from warmer, more arid environments are longer lived in storage (Walters et al., 2005; Probert et al., 2009). This could be a factor of growing season length, whereby alpine species have a condensed window of time for seed maturation and immature seeds lose viability more quickly (Sanhewe and Ellis, 1996; Mondoni et al., 2011; Hay and Probert, 2013); however, other mechanisms could also result in the shorter longevity of alpine plants in storage.

Many factors can influence seed storage behavior, such as seed structure, composition, maturity, size, dormancy type, moisture content, and vigor (Justice and Bass, 1978; Walters, 2004; Tausch et al., 2019). Endospermic and dormant seeds have been shown to be shorter lived than those without endosperm and those that are nondormant (Tausch et al., 2019). As such, the endosperm and embryo characteristics of the species in the present study were explored using “The Comparative Internal Morphology of Seeds” (Martin, 1946). Seeds of *Castilleja* have dwarf embryos, those of *Heterotheca* and *Saussurea* have spatulate embryos with no appreciable amount of endosperm, and *Physaria* have bent embryos without endosperm. In general, the difference in longevity among these four species was not large enough to draw any significant biological conclusions around the role of endosperm and dormancy in seed longevity. Shorter longevity could also be a factor of seed mass and elevation (Satyanti et al., 2018). Due to the limited number of species from the same elevation and no significant difference in seed longevity, seed mass was not explored in the context of this study. Further comparative studies involving seeds of different sizes and sourced from a range of elevations would need to be performed to understand these drivers in the context of Colorado flora.

Although this study used artificial aging to show that alpine species from Colorado are short lived, their seeds may survive for years to decades in ex‐situ seed bank conditions, in which temperature and relative humidity are kept low. The standard protocol for seeds stored in ex‐situ seed banks is to test viability every 5–10 years (FAO/IPGRI, 1994; FAO, 2014). At Denver Botanic Gardens, we test the viability of our seed collections every five years; however, given the results of this study, we will amend our protocols to test the viability of stored alpine seeds every three years (Hay and Probert, 2013; Satyanti et al., 2018). This will allow us to empirically test the seed longevity of alpine species and provide a more accurate understanding of predicted loss of viability in seed bank conditions. Artificial aging tests were vital in elucidating that Colorado alpine species may be short lived in seed banks because they provide early and expedited information, which will allow us to better manage and adjust conservation protocols. Artificial aging tests should be performed on species for which there is limited longevity information, on numerous species from one ecosystem, or on multiple populations of one species, to better understand the influence of climate, habitat, and phylogenetics on seed longevity, and on species for which exceptionality status is not known. This information can then be applied to congeners from comparable climatic areas worldwide, for which seed storage behavior is likely similar (e.g., Probert et al., 2009; Mondoni et al., 2011). Performing more artificial aging tests will provide more longevity information, which will lead to more accurate species‐specific or biome‐specific storage protocols.

Seed banking has become the primary method of ex‐situ conservation, and reports such as the Global Strategy for Plant Conservation and the North American Botanic Garden Strategy for Plant Conservation have urged conservationists to safeguard the world's flora through ex‐situ collections (Convention on Biological Diversity, 2012; BGCI, 2016). More specifically, the recently published North American Botanic Garden Strategy for Alpine Plant Conservation (the Strategy) has advocated for the conservation of alpine plants and ecosystems, as alpine areas are particularly threatened by climate change (Intergovernmental Panel on Climate Change, 2014; Ripley et al., 2020). Two targets within the Strategy call for the ex‐situ conservation of alpine plants—60% of all alpine plants and 75% of threatened alpine plants—by 2030. These targets involve important actions in the process toward alpine plant conservation; however, if seeds go into storage with little understanding of their longevity, time and resources may be wasted if viability is lost prior to utilizing the seeds. As such, seeds should not be collected haphazardly with no plans for use in the subsequent decade. Seed conservation measures for alpine plants should involve viability testing every three years, grow‐outs in greenhouses, and the reintroduction and/or recollection of the seeds if a decline in viability is becoming evident.

Through this study, we have some evidence that five alpine plants of Colorado are short lived, which has implications for their ex‐situ conservation storage in seed banks. The method of artificial aging provided important knowledge about the potential longevity of these species, which will allow us to effectively manage seed collections of alpine plants. Seeds that are short lived in storage may be some of the most difficult collections to manage, as the collections need to be utilized within a shorter period after collection to avoid wasting resources. Alternative conservation methods could be considered, such as the cryopreservation of the seeds; however, more empirical evidence about alpine seed longevity is needed to understand the benefit of this method. For now, the viability of alpine collections should be tested every few years with a short‐term plan for how the seeds will be used. The Colorado alpine ecosystem is relatively pristine, but is becoming more vulnerable. Rare alpine endemics are particularly at risk, and conservation actions are needed to protect these valuable species. Effective ex‐situ conservation measures rely on knowledge of germination requirements and longevity information. This study is an important step in providing that information for the rare alpine treasures of Colorado.

## Supporting information

Appendix S1. Data set of raw germination results.

Appendix S2. Data set of raw seed longevity results.

## Data Availability

The raw data from the study are available in the Supporting Information as Appendices S1 and S2.

## APPENDIX 1 Voucher information for species used in this study. All vouchers are housed in the Kathryn Kalmbach Herbarium (KHD) at the Denver Botanic Gardens (Denver, Colorado, USA)

**Table:**

Species	Voucher specimen accession no.	Geographic coordinates	Collection locality
*Castilleja puberula* Rydb.	KHD00067933	40.09732, –105.58577	Mt. Audubon
*Castilleja puberula*	KHD00068434	39.592395, –105.724728	Guanella Pass
*Heterotheca pumila* (Greene) Semple	KHD00067948	39.369878, –106.080295	Hoosier Pass
*Ipomopsis globularis* (Brand) W. A. Weber	KHD00067947	39.1315, –106.182299	Weston Pass
*Physaria alpina* Rollins	KHD00068703	39.207167, –106.165989	Fourmile Creek Trail
*Physaria alpina*	KHD00071330	39.13044, –106.1837	Weston Pass
*Saussurea weberi* Hultén	KHD00067949	39.370626, –106.081579	Hoosier Pass
